# 

**Published:** 1871-10

**Authors:** 


### ARTICLE IV.

### *Virginia State Dental Association.*

The second annual meeting of the Virginia State Dental Association, will be held in Richmond, on the first Thursday of November. *All* members of the profession throughout the *entire* State are *earnestly* requested to attend, as business of importance requiring strict attention will be presented to the convention for its action.

GEO. F. KEESEE, Sec'y.

*Virginia State Dental Association.*—We are requested to urge upon our professional brethren of Virginia, the necessity existing for a full attendance at the meeting to be held in Richmond on the first Thursday of November next, not only of the members but of all practicing dentists in the State, as the vital interests of the profession demand a thorough establishment of the Association on a sure foundation. We trust, therefore, that this call will be attended to on the part of all who have the good of their profession at heart, and that the meeting may in every respect accomplish the purpose for which it is called.

